# Supplementary material for: Climate: The dominant factor influencing the spatial distribution pattern of the leaf trait network of Populus euphratica along the main stream of the Tarim River
Source: PLoS One. 2025 May 7;20(5):e0323305. doi: 10.1371/journal.pone.0323305 (PMC12057974; doi:10.1371/journal.pone.0323305)
Supplement: S1 File — (ZIP) [file pone.0323305.s001.zip › Supplemental information/S1 Table.docx]

**S1 Table. Sample point information.**

| **Sample point** | **Longitude/ °E** | **Latitude/ °N** | **Altitude/ m** | **Distance from bank/ km** |
| --- | --- | --- | --- | --- |
| T1 | 80.916 | 41.137 | 1084 | 18.475 |
| T2 | 80.944 | 40.437 | 1025 | 1.093 |
| T3 | 81.155 | 40.425 | 1018 | 9.569 |
| T4 | 81.6 | 40.757 | 1001 | 2.982 |
| T5 | 81.929 | 40.654 | 994 | 0.828 |
| T6 | 82.152 | 40.845 | 988 | 0.614 |
| T7 | 82.343 | 41.053 | 978 | 2.954 |
| T8 | 82.743 | 40.963 | 970 | 0.261 |
| T9 | 83.015 | 40.904 | 962 | 0.969 |
| T10 | 83.162 | 41.052 | 957 | 0.774 |
| T11 | 83.316 | 41.006 | 955 | 0.993 |
| T12 | 84.202 | 41.3 | 932 | 3.596 |
| T13 | 84.213 | 41.245 | 934 | 0.957 |
| T14 | 84.238 | 41.172 | 933 | 0.524 |
| T15 | 85.127 | 41.994 | 974 | 4.346 |
| T16 | 86.163 | 41.19 | 891 | 0.171 |
| T17 | 86.843 | 40.924 | 874 | 1.85 |
| T18 | 87.076 | 40.894 | 868 | 12.269 |
| T19 | 87.541 | 40.645 | 856 | 4.994 |
| T20 | 87.904 | 40.454 | 845 | 0.045 |
