# Supplementary material for: Climate: The dominant factor influencing the spatial distribution pattern of the leaf trait network of Populus euphratica along the main stream of the Tarim River
Source: PLoS One. 2025 May 7;20(5):e0323305. doi: 10.1371/journal.pone.0323305 (PMC12057974; doi:10.1371/journal.pone.0323305)
Supplement: S1 File — (ZIP) [file pone.0323305.s001.zip › Supplemental information/S2 Table.docx]

**S2 Table. Classification units and abbreviations for leaf traits.**

| **Classification** | **Full name** | **Unit** | **Abbreviation** |
| --- | --- | --- | --- |
| Leaf morphological traits | Leaf length | cm | LL |
|  | Leaf width | cm | LW |
|  | Leaf area | cm^2^ | LA |
|  | Leaf dry weight | g | LDW |
|  | Leaf water content | % | LWC |
|  | Leaf dry matter content | g | LDMC |
|  | Specific leaf area | cm^2^/g | SLA |
|  | Leaf thickness | μm | LT |
| Stoichiometric characteristics | Leaf total organic matter | g/kg | LC |
|  | Leaf total nitrogen | g/kg | LN |
|  | Leaf total phosphorus | g/kg | LP |
|  | Leaf total potassium | g/kg | LK |
|  | Leaf organic matter : nitrogen ratio | \ | LC:N |
|  | Leaf organic matter : phosphorus ratio | \ | LC:P |
|  | Leaf nitrogen : phosphorus ratio | \ | LN:P |
| Leaf anatomical structure traits | Sponge tissue | μm | ST |
|  | Upper epidermis thickness | μm | UE |
|  | Lower epidermis thickness | μm | LE |
|  | Mucilage cells | μm^2^ | MC |
|  | Palisade tissue | μm | PT |
|  | Upper stratum corneum | μm | USC |
|  | Lower stratum corneum | μm | LSC |
|  | Midvein vascular bundle | μm^2^ | MVB |
|  | Sclerenchyma | μm^2^ | SC |
|  | ratio of palisade tissue to sponge tissue | % | P/S |
|  | Cell tension ratio | % | CTR |
|  | Spongy ratio | % | SR |
