# Supplementary material for: Climate: The dominant factor influencing the spatial distribution pattern of the leaf trait network of Populus euphratica along the main stream of the Tarim River
Source: PLoS One. 2025 May 7;20(5):e0323305. doi: 10.1371/journal.pone.0323305 (PMC12057974; doi:10.1371/journal.pone.0323305)
Supplement: S1 File — (ZIP) [file pone.0323305.s001.zip › Supplemental information/S3 Table.docx]

**S3 Table. Soil factors.**

| **Full name** | **Unit** | **Abbreviation** |
| --- | --- | --- |
| Water content | % | WC |
| Electrical conductivity | μs/cm | EC |
| Total salt | g/kg | TS |
| pH | \ | pH |
| Soil total organic matter | g/kg | SOM |
| Soil total nitrogen | g/kg | STN |
| Soil total phosphorus | g/kg | STP |
| Soil total potassium | g/kg | STK |
| Soil organic matter : nitrogen ratio | \ | SC:N |
| Soil organic matter : phosphorus ratio | \ | SC:P |
| Soil nitrogen : phosphorus ratio | \ | SN:P |
