# Supplementary material for: Climate: The dominant factor influencing the spatial distribution pattern of the leaf trait network of Populus euphratica along the main stream of the Tarim River
Source: PLoS One. 2025 May 7;20(5):e0323305. doi: 10.1371/journal.pone.0323305 (PMC12057974; doi:10.1371/journal.pone.0323305)
Supplement: S1 File — (ZIP) [file pone.0323305.s001.zip › Supplemental information/S4 Table.docx]

**S4 Table. Climate factors.**

| **Climate factors** | **Full name** |
| --- | --- |
| BIO1 | Annual mean temperature |
| BIO2 | Mean diurnal range (mean of monthly (max temp - min temp)) |
| BIO3 | Isothermality (BIO2/BIO7) (×100) |
| BIO4 | Temperature seasonality (standard deviation ×100) |
| BIO5 | Max temperature of warmest month |
| BIO6 | Min temperature of coldest month |
| BIO7 | Temperature annual range (BIO5-BIO6) |
| BIO8 | Mean temperature of wettest quarter |
| BIO9 | Mean temperature of driest quarter |
| BIO10 | Mean temperature of warmest quarter |
| BIO11 | Mean temperature of coldest quarter |
| BIO12 | Annual precipitation |
| BIO13 | Precipitation of wettest month |
| BIO14 | Precipitation of driest month |
| BIO15 | Precipitation seasonality (coefficient of variation) |
| BIO16 | Precipitation of wettest quarter |
| BIO17 | Precipitation of driest quarter |
| BIO18 | Precipitation of warmest quarter |
| BIO19 | Precipitation of coldest quarter |
