# Supplementary material for: Climate: The dominant factor influencing the spatial distribution pattern of the leaf trait network of Populus euphratica along the main stream of the Tarim River
Source: PLoS One. 2025 May 7;20(5):e0323305. doi: 10.1371/journal.pone.0323305 (PMC12057974; doi:10.1371/journal.pone.0323305)
Supplement: S1 File — (ZIP) [file pone.0323305.s001.zip › Supplemental information/S5 Table.docx]

**S5 Table. Network parameters of 20 sampling points.**

| **Sample point** | **Average path length** | **Diameter** | **Edge density** | **Average clustering coefficient** | **Modularity** |
| --- | --- | --- | --- | --- | --- |
| T1 | 1.422 | 3.731 | 0.149 | 0.531 | 0.58 |
| T2 | 1.882 | 4.697 | 0.123 | 0.464 | 0.492 |
| T3 | 1.802 | 3.871 | 0.114 | 0.659 | 0.674 |
| T4 | 1.923 | 5.176 | 0.165 | 0.681 | 0.39 |
| T5 | 1.523 | 3.39 | 0.247 | 0.712 | 0.265 |
| T6 | 2.24 | 5.837 | 0.146 | 0.508 | 0.529 |
| T7 | 1.907 | 4.307 | 0.108 | 0.434 | 0.673 |
| T8 | 4.595 | 11.17 | 0.129 | 0.621 | 0.508 |
| T9 | 1.643 | 3.333 | 0.178 | 0.439 | 0.421 |
| T10 | 2.91 | 7.796 | 0.123 | 0.491 | 0.536 |
| T11 | 1.939 | 5.479 | 0.15 | 0.529 | 0.498 |
| T12 | 2.483 | 5.814 | 0.117 | 0.462 | 0.641 |
| T13 | 2.297 | 4.821 | 0.138 | 0.519 | 0.616 |
| T14 | 2.546 | 5.791 | 0.101 | 0.466 | 0.584 |
| T15 | 2.028 | 5.212 | 0.103 | 0.48 | 0.668 |
| T16 | 2.286 | 5.331 | 0.102 | 0.478 | 0.636 |
| T17 | 2.571 | 6.173 | 0.127 | 0.533 | 0.606 |
| T18 | 2.037 | 4.537 | 0.156 | 0.465 | 0.507 |
| T19 | 1.615 | 3.758 | 0.126 | 0.727 | 0.644 |
| T20 | 1.343 | 3.09 | 0.11 | 0.511 | 0.639 |
| MAX | 4.595 | 11.17 | 0.247 | 0.727 | 0.674 |
| MIN | 1.343 | 3.09 | 0.101 | 0.434 | 0.265 |
| AVERAGE | 2.15 | 5.166 | 0.136 | 0.535 | 0.555 |
