# Supplementary material for: Climate: The dominant factor influencing the spatial distribution pattern of the leaf trait network of Populus euphratica along the main stream of the Tarim River
Source: PLoS One. 2025 May 7;20(5):e0323305. doi: 10.1371/journal.pone.0323305 (PMC12057974; doi:10.1371/journal.pone.0323305)
Supplement: S1 File — (ZIP) [file pone.0323305.s001.zip › Supplemental information/S7 Table.docx]

**S7 Table. Relationship between network node closeness and environmental factors.**

*: At *P*<0.05 (two-tailed), the correlation was significant. **: At *P*<0.01 (two-tailed), the correlation was significant.

| **Trait** | **BIO3** | **BIO6** | **BIO13** | **SWC%** | **STK(g/kg)** | **SOM(g/kg)** |
| --- | --- | --- | --- | --- | --- | --- |
| LL | / | / | / | / | / | / |
| LDW | / | / | / | / | / | / |
| LT | / | -.456* | / | / | / | / |
| LW | / | / | / | / | / | / |
| LA | / | / | / | / | / | / |
| LDMC | / | / | -.586** | / | / | / |
| SLA | / | / | / | / | / | / |
| LWC | / | / | -.578** | / | / | / |
| LSC | .512* | / | / | / | / | / |
| USC | / | / | / | / | / | / |
| MC | / | / | / | / | / | / |
| MVB | / | / | / | / | / | / |
| P/S | / | / | / | / | / | / |
| PT | / | -.526* | / | / | / | / |
| SR | / | / | / | / | / | / |
| ST | / | / | / | / | / | / |
| TWT | / | / | -.446* | / | / | / |
| LE | / | / | / | / | / | / |
| UE | / | / | / | / | / | / |
| CTR | / | / | / | / | / | / |
| LC | / | / | / | / | / | / |
| LC:N | / | / | / | / | / | / |
| LC:P | / | -.646** | -.492* | / | / | / |
| LK | / | / | / | / | / | / |
| LN | / | / | / | / | / | / |
| LN:P | / | / | / | / | / | / |
| LP | / | -.497* | / | / | / | / |
