# Supplementary material for: Climate: The dominant factor influencing the spatial distribution pattern of the leaf trait network of Populus euphratica along the main stream of the Tarim River
Source: PLoS One. 2025 May 7;20(5):e0323305. doi: 10.1371/journal.pone.0323305 (PMC12057974; doi:10.1371/journal.pone.0323305)
Supplement: Attachment — Submitted filename: PONE-D-24-52929_review.docx [file pone.0323305.s002.docx]

**Overall summary and opinion:**

Plant functional traits, particularly leaf traits, play a critical role in understanding how plants adapt to environmental changes and maintain ecosystem functions. Leaf traits serve as a bridge between plants and their environment, reflecting adaptability, self-regulation, and photosynthetic capacity. Variations in these traits, influenced by environmental stressors and phylogenetic history, reveal the strategies plants use to optimize resource acquisition, utilization, and distribution. As global environmental changes intensify, research on the relationship between leaf traits and environmental factors is essential for uncovering plant survival strategies and their ecological adaptation mechanisms. This knowledge not only enhances our understanding of plant-environment interactions but also supports the conservation and management of ecosystems.

This study focuses on *Populus euphratica*, a drought-resistant species found along the ecologically fragile Tarim River in Xinjiang, characterized by water scarcity, severe soil erosion, and harsh climatic conditions. By integrating leaf trait network analysis, principal component analysis (PCA), and variance decomposition analysis (VPA), researchers explore how *P. euphratica* optimizes its leaf traits to adapt to the challenging environment. Building on a prior study that identified key traits like the lower stratum corneum (LSC) and midvein vascular bundle (MVB) as central to drought resistance, this study further examines the spatial variability of leaf traits across the Tarim River basin and their relationship with environmental factors like soil and climate. It is broadly understood that the goal is to reveal *P. euphratica*'s adaptive strategies, providing insights for protecting these vital riparian forests and sustaining their role in mitigating desertification and supporting oasis ecosystems.

Towards this end I would like to recommend a revised resubmission of the manuscript by considering following changes to the manuscript

**Major changes / revisions for authors:**

- One of the major concerns for this manuscript is the lack of originality in the study design as well as the data set described. The dataset used in this manuscript is described and analyzed using similar methods in [**https://doi.org/10.3390/f15030437**](https://doi.org/10.3390/f15030437) by the same authors. The LTN analysis does not significantly advance the ideas or methodology described in the said manuscript. It is recommended that the authors explicitly state that this study is an extension of the said previously published work.
- It is helpful for the readers to know at some point why authors decide to use LTN strategies rather than directly investigate measured leaf traits. Therefore authors need to present a clear account of how measured leaf traits directly associate with soil and environmental data or present clear evidence that this was investigated in a previous study.
- In order to alleviate some of the shortcomings described above, I personally feel that the authors should establish the unique space in which findings of this paper can be highlighted by focusing on the climate and environmental variables that are described in this study. They must first establish the environmental clines or at the least fully characterize the environmental and soil data and their variation along the collection points which can then be attributed to the LTN strategies. It is difficult to follow the main points in this manuscript due to the fragmented nature in which results are described. The manuscript would benefit from presenting a digestion of the data and LTN strategies rather than present a mere descriptive account of associations and statistics in the discussion section.
- PC1 and PC2 describes a large portion of the variance across collection points using climatic variables. It also clear that these climatic variables influence LTN strategies in a collective manner. Therefore, it may benefit this study to look at the correlation of PCs to the LTN characteristics as well as individual climatic variables. A clustering of the collection points in terms of climatic and soil variables is absent in the study and such a characterization is fundamentally important in trying to understand ecosystem dynamics of this riparian forest.
- The statistical analysis presented here in this study is weaker than even the authors’ first publication on the same dataset. For example, for statistics described (CV) in Figure 3 and 5, it is required to report at the least bootstrap standard error values. It is helpful for readers to know some measure of confidence in these summary stats especially given that there are only 20 sampling points in this study. Another example is that authors do not report the statistical significance of the variance component estimates described in Figure 10. One can use a method of permutation testing under full and reduced models using the same vegan R-package to do this with ease. There seems to be a general lack of disregard to statistical rigor shown throughout the manuscript. For example, claims made in lines 307-309 cannot be supported using statistical evidence. Therefore, it is strongly suggested that this is remedied in the revised submission.

**Minor changes / suggestions for authors:**

- Lines 66-76: This entire section contains repetitive words and ideas. It is not entirely clear what the authors are trying to convey in this section as the sentences are too long and convoluted.
- Lines 104-106: Please cite the ‘few reports’ mentioned in this section. This is especially required since it sets up the premise for this manuscript. Mention how this investigation can be set apart from a very similar analysis carried out by Yao et al., 2024 cited in this manuscript since the methodology and the population in question are the same.
- Line 136: Should be DBH range 20-30cm instead of 20>30 cm?
- Line 155: This notation for range is unconventional. Numbers should be separated by a hyphen or as ranges. For example, [0, 20] or (0, 20]. First indicates that the zero and twenty are included and the second notation states that the range includes values greater than zero up to and including twenty.
- Figure 3: It will be helpful for the readers if colors in the sub-figure (a) match that of (b). Although I presume that shades of green were used in this figure since they depict attributes of green leaves, I feel a more color-blind friendly scheme would be more appropriate here.
