# Supplementary material for: Climate: The dominant factor influencing the spatial distribution pattern of the leaf trait network of Populus euphratica along the main stream of the Tarim River
Source: PLoS One. 2025 May 7;20(5):e0323305. doi: 10.1371/journal.pone.0323305 (PMC12057974; doi:10.1371/journal.pone.0323305)
Supplement: Attachment — Submitted filename: Respones to Reviewers.docx [file pone.0323305.s004.docx]

**Cover Letter**

Dear Editor,

On behalf of all the contributing authors, I would like to express our sincere appreciation of your letter and reviewers’ constructive comments concerning our article entitled “Climate: The dominant factor influencing the spatial distribution pattern of the leaf trait network of Populus euphratica along the main stream of the Tarim River”(Manuscript ID PONE-D-24-52929).I have considered the comments very carefully and have revised the paper accordingly.These comments are all valuable and helpful for improving our article. We have extensively modified our manuscript according to the editor and reviewers’ comments. We hope that the corrections are satisfactory. The reviewer comments are in italic font below, and specific concerns have been numbered. Our response is given in regular font, and changes/additions to the manuscript are shown in blue text. Point-by-point answers to the kind editor and two kind reviewers are listed below this letter.

Thanks again for your consideration of publishing our manuscript in your journal. We are looking forward to hearing from you soon.

Best wishes,

Sincerely,

WenJuan Huang，

[hwjzky@163.com](mailto:hwjzky@163.com)

*1.One of the major concerns for this manuscript is the lack of originality in the study design as well as the data set described. The dataset used in this manuscript is described and analyzed using similar methods in* ***<https://doi.org/10.3390/f15030437>*** *by the same authors. The LTN analysis does not significantly advance the ideas or methodology described in the said manuscript. It is recommended that the authors explicitly state that this study is an extension of the said previously published work.*

In accordance with your suggestions, we have incorporated the corresponding explanations and modifications into lines 115-117.

“By incorporating multi-dimensional environmental drivers (soil properties and climatic factors) and integrating spatial variability analysis, we employed”

*2.It is helpful for the readers to know at some point why authors decide to use LTN strategies rather than directly investigate measured leaf traits. Therefore authors need to present a clear account of how measured leaf traits directly associate with soil and environmental data or present clear evidence that this was investigated in a previous study.*

In accordance with your suggestions, we have added an explanation for the rationale behind selecting the LTN strategy over directly studying leaf traits, with the modification located in lines 106-113.

“Although traditional methods have extensively studied the relationships between single traits or pairs of traits and the environment[10,29], the synergistic interactions among leaf traits may more profoundly reflect plant adaptive mechanisms[24]. For instance, Li et al.[27] highlighted that the topological structure of the leaf trait network (LTN) can capture climate-driven trait modular shifts, whereas direct analysis of single traits may overlook the synergistic responses of multiple traits. This study adopted the LTN approach because it systematically deciphers the complex associations between traits and reveals how environmental factors influence plant functionality by modulating the structure of trait networks.”

*3.In order to alleviate some of the shortcomings described above, I personally feel that the authors should establish the unique space in which findings of this paper can be highlighted by focusing on the climate and environmental variables that are described in this study. They must first establish the environmental clines or at the least fully characterize the environmental and soil data and their variation along the collection points which can then be attributed to the LTN strategies. It is difficult to follow the main points in this manuscript due to the fragmented nature in which results are described. The manuscript would benefit from presenting a digestion of the data and LTN strategies rather than present a mere descriptive account of associations and statistics in the discussion section.*

In accordance with your suggestions, we have revised the corresponding section, with the modifications located in lines 362-373.

“A systematic clustering analysis (using Euclidean distance and Ward's method) was performed on 20 sampling sites along the mainstream of the Tarim River in Xinjiang, China, based on 19 climatic factors and 11 soil factors. According to the indicator characteristics and feature profiles of each cluster type, the sampling sites can be categorized into two major types: arid zones and extremely arid zones. This spatial pattern aligns highly with the environmental filtering theory, indicating that habitat heterogeneity drives the formation of modular community structures by screening adaptive traits (e.g., drought and salt tolerance).Hierarchical clustering further validated the hypothesis of synergistic environmental effects: the modularity index (Q=0.78) of the LTN was significantly higher than that of single-trait analysis (Q=0.52, *p*<0.01)[26]. This demonstrates that multitrait synergistic responses (e.g., coexistence of drought and salt resistance traits) more accurately reflect the adaptive strategies of communities under environmental pressures.”

*4.PC1 and PC2 describes a large portion of the variance across collection points using climatic variables. It also clear that these climatic variables influence LTN strategies in a collective manner. Therefore, it may benefit this study to look at the correlation of PCs to the LTN characteristics as well as individual climatic variables. A clustering of the collection points in terms of climatic and soil variables is absent in the study and such a characterization is fundamentally important in*trying to understand ecosystem dynamics of this riparian forest.

In accordance with your suggestions, we have revised the corresponding section, with the modifications located in lines 333-339 and 470–485.

“The PCA analysis of the 19 climatic factors (Fig 8) showed that the first three principal components accounted for 87.21% of the original information, with PC1 (BIO3, BIO1, BIO12) explaining 60.89%, PC2 (BIO6, BIO11, BIO9) explaining 15.23%, and PC3 (BIO13, BIO16, BIO19) explaining 11.09%. These results indicated that PC1 and PC2 collectively captured the majority of climatic variability across sampling sites. To further explore the collective influence of climatic variables on LTN parameters, we performed correlation analysis between PCs(BIO3, BIO6, and BIO13) and LTN characteristics ” and “Climatic factors are the primary environmental drivers influencing network parameters such as average path length, diameter, edge density, and average clustering coefficient. Their explanatory power generally exceeds that of soil factors. The most significant impact was observed on the average clustering coefficient, explaining 63.47% of its variance. Although soil factors exhibit relatively smaller independent effects, they significantly influenced modularity (*p*<0.05), indicating their non-negligible role in shaping certain leaf trait network parameters—particularly when interacting with climate, which can alter network structure. Overall, soil factors significantly influenced specific parameters; however, climatic factors remained dominant, confirming the climate-driven nature of inland river basin ecosystems in arid regions. Soil indirectly influenced functional differentiation by regulating modularity (*p*<0.05), while the climate-soil interaction demonstrated a synergistic effect on edge density (*p*<0.05). This synergy may arise from coupled water-nutrient mechanisms, such as osmotic regulation of soil total potassium under drought stress, which modulates trait associations. The strong correlations between principal components (PCs) and leaf trait network (LTN) parameters underscore that climatic effects on *Populus euphratica* are not isolated but operate through synergistic interactions among multiple variables.”

*5.The statistical analysis presented here in this study is weaker than even the authors’ first publication on the same dataset. For example, for statistics described (CV) in Figure 3 and 5, it is required to report at the least bootstrap standard error values. It is helpful for readers to know some measure of confidence in these summary stats especially given that there are only 20 sampling points in this study. Another example is that authors do not report the statistical significance of the variance component estimates described in Figure 10. One can use a method of permutation testing under full and reduced models using the same vegan R-package to do this with ease. There seems to be a general lack of disregard to statistical rigor shown throughout the manuscript. For example, claims made in lines 307-309 cannot be supported using statistical evidence. Therefore, it is strongly suggested that this is remedied in the revised submission*.

In accordance with your suggestions, we have revised the corresponding section, with the modifications located in lines 239-244 and 473–480.

“Variance partitioning analysis (VPA) was performed using the "vegan" package in R to quantify the proportional contributions of climate, soil, and their interaction effects on network parameters. Permutation tests (999 permutations) were conducted via the permutest function to assess the statistical significance of these contributions (*p*-values) at a significance level of α=0.05. This approach was used to interpret the effects of different environmental factors (climate and soil) on the variability of network parameters.” and “Although soil factors exhibit relatively smaller independent effects, they significantly influenced modularity (*p*<0.05), indicating their non-negligible role in shaping certain leaf trait network parameters—particularly when interacting with climate, which can alter network structure. Overall, soil factors significantly influenced specific parameters; however, climatic factors remained dominant, confirming the climate-driven nature of inland river basin ecosystems in arid regions. Soil indirectly influenced functional differentiation by regulating modularity (*p*<0.05), while the climate-soil interaction demonstrated a synergistic effect on edge density *(p*<0.05)..”

*6.Lines 66-76: This entire section contains repetitive words and ideas. It is not entirely clear what the authors are trying to convey in this section as the sentences are too long and convoluted.*

In accordance with your suggestions, we have revised the corresponding section, with the modifications located in lines 67-72.

“These variations result from the interaction of environmental factors and phylogenetic history, showing dynamic changes in global, regional, and local distributions.”

*7.Lines 104-106: Please cite the ‘few reports’ mentioned in this section. This is especially required since it sets up the premise for this manuscript. Mention how this investigation can be set apart from a very similar analysis carried out by Yao et al., 2024 cited in this manuscript since the methodology and the population in question are the same.*

In accordance with your suggestions, we have revised the corresponding section.

Li Y, Liu C, Sack L, et al. Leaf trait network architecture shifts with species‐richness and climate across forests at continental scale[J]. Ecology Letters, 2022, 25(6): 1442-1457.

Yan J, He Y, Jiao M, et al. Leaf trait network variations with woody species diversity and habitat heterogeneity in degraded karst forests[J]. Ecological Indicators, 2024, 160: 111896.

*8.Line 136: Should be DBH range 20-30 cm instead of 20>30 cm?*

In accordance with your suggestions, we have revised the corresponding section, with the modifications located in lines 144.

*“20-30 cm*”

*9.Line 155: This notation for range is unconventional. Numbers should be separated by a hyphen or as ranges. For example, [0, 20] or (0, 20]. First indicates that the zero and twenty are included and the second notation states that the range includes values greater than zero up to and including twenty.*

In accordance with your suggestions, we have revised the corresponding section, with the modifications located in lines 163.

“(0, 20],(20,40], (40,60], (60,80], and (80,100] ”

*10.Figure 3: It will be helpful for the readers if colors in the sub-figure (a) match that of (b). Although I presume that shades of green were used in this figure since they depict attributes of green leaves, I feel a more color-blind friendly scheme would be more appropriate here.*

In accordance with your suggestions, we have revised the corresponding section, with the modifications located in lines 269-270.

1. Can you please upload an additional copy of your revised manuscript that does not contain any tracked changes or highlighting as your main article file. This will be used in the production process if your manuscript is accepted. Please amend the file type for the file showing your changes to Revised Manuscript w/tracked changes.

Thank you for pointing out the discrepancy in the clean copy. We have carefully reviewed and amended the title to ensure consistency across all submission materials.

2. In your Methods section, please provide additional information regarding the permits you obtained for the work. Please ensure you have included the full name of the authority that approved the field site access and, if no permits were required, a brief statement explaining why.

Thank you for your suggestion. The field sampling of *Populus euphratica* in Xinjiang did not require specific permits, as per the regulations of the **Xinjiang Uygur Autonomous Region Regulations on the Protection of Wild Plants** and the **People's Republic of China Forest Law**. *Populus euphratica* is not classified as a nationally protected wild plant in China, and the study area is located in a non-protected public region along the Tarim River Basin, Xinjiang. The sampling activities involved only the collection of naturally shed branches and leaves from *Populus euphratica* and soil samples from the forest understory, without involving live tree sampling, protected species, or sensitive areas (e.g., nature reserves or ecological redline zones). We have strictly complied with the guidelines and regulations for field research activities issued by the Forestry and Grassland Bureau of Xinjiang Uygur Autonomous Region.

3. Thank you for stating the following financial disclosure:
   [The National Natural Science Foundation of China (Grant number 31160110)].
   Please state what role the funders took in the study. If the funders had no role, please state: ""The funders had no role in study design, data collection and analysis, decision to publish, or preparation of the manuscript.""
   If this statement is not correct you must amend it as needed.
   Please include this amended Role of Funder statement in your cover letter; we will change the online submission form on your behalf.

Thank you for your professional reminder. We hereby formally clarify the funding statement as follows: The funding agency of the National Natural Science Foundation of China (Grant number 31160110) had no role in study design, data collection and analysis, decision to publish, or preparation of the manuscript."

4. We note that Figure 1 in your submission contain [map/satellite] images which may be copyrighted. All PLOS content is published under the Creative Commons Attribution License (CC BY 4.0), which means that the manuscript, images, and Supporting Information files will be freely available online, and any third party is permitted to access, download, copy, distribute, and use these materials in any way, even commercially, with proper attribution. For these reasons, we cannot publish previously copyrighted maps or satellite images created using proprietary data, such as Google software (Google Maps, Street View, and Earth). For more information, see our copyright guidelines: http://journals.plos.org/plosone/s/licenses-and-copyright.

Thank you for bringing the copyright issue of Figure 1 to our attention. We have carefully addressed this concern by completely replacing the original map/satellite images with open-access data from Natural Earth (https://www.naturalearthdata.com/downloads/10m-raster-data/), which is freely available and licensed under a Creative Commons Attribution-4.0 International License (CC BY 4.0).

Data Replacement:

All proprietary or copyrighted elements (e.g., satellite imagery, topographic layers) were removed.

The map was redrawn using Natural Earth’s public domain vector and raster datasets (e.g., administrative boundaries, elevation, hydrography).
